# Editorial: Psychological factors in physical education and sport, volume II

**DOI:** 10.3389/fpsyg.2023.1202761

**Published:** 2023-06-13

**Authors:** Manuel Gómez-López, Carla Chicau Borrego, Marianna Alesi

**Affiliations:** ^1^Department of Physical Activity and Sport, Faculty of Sports Sciences, University of Murcia, Murcia, Spain; ^2^Research Center in Life Quality, Sport Sciences School of Rio Maior, Polytechnic Institute of Santarém, Rio Maior, Portugal; ^3^Department of Psychology, Educational Sciences, and Human Movement, University of Palermo, Palermo, Italy

**Keywords:** motivation, education, sport, physical education, psychological wellbeing

This Research Topic compiles the most recent research on some of the factors that influence the physical and psychological wellbeing of those who practice physical-sports activities and how to promote adherence in both the educational and sports contexts.

In recent years, studies on athlete leadership have increased. Today it is recognized as a vital component of team performance and as a determining factor that facilitates a more positive and sustainable sporting environment. The results of the study developed by Kim and Cruz showed that effective leadership in sport depends on the interaction between the leadership behaviors of the coach, the personal characteristics of the players and situational factors. Furthermore, the importance of transformational leadership was also demonstrated as an important requirement for creating a more positive and sustainable sport environment. In this group, we find studies that highlight the importance of the coach, and more specifically of leadership styles and their relationship with resilience or cohesion in athletes. In this group, we find studies that highlight the importance of the coach, and more specifically of leadership styles and their relationship with resilience or cohesion in athletes. The study by Liu et al. recommends that elite youth soccer schools pay attention to leadership styles and apply them to sports practice. On the other hand, Flemington et al., suggest that a cohesive team shares leadership responsibilities with many peer bonds. In addition to this, there is also the need to develop and validate a scale that can measure the leadership of the coaching service (Takamatsu).

On the other hand, we have perceptions and their importance as determinants of people's behavior. In this field, not much is known about athletes' perceptions of their sport and the links of these perceptions with the physical and psychological benefits perceived by athletes, such as sport commitment or quality of life. In general, it has been shown that athletes' perceptions seem to be relevant for experiencing training satisfaction and general wellbeing. As an example, we have the results provided by Limpo et al.. This study revealed that karateka's perceived benefits of karate predicted engagement directly and quality of life indirectly through vigor. In general, it was shown that karateka's perceptions appeared to be relevant to experiencing training satisfaction and general wellbeing. Another perception that influences sport performance is the motivational climate generated by the coach during training and competitions. Specifically, it has been shown that a mastery motivational climate reinforces in the athlete the perception of performance as the result of task mastery and personal effort, as opposed to a competitive climate that reinforces competition and rivalry among team members. The study developed by Appleton et al. suggested that the EDMCQ-C scale can be used to provide meaningful latent mean comparisons of the empowering and disempowering climates created by coaches among athletes.

Other key factors in sport performance, are emotional intelligence, confidence, attention, and cognitive reappraisal, since sport is an emotional experience. Mercader-Rubio et al. demonstrated that emotional intelligence is a predictor of identified regulation, introjected regulation and external regulation. In addition, Wang D. et al. concluded that the development of sport confidence and mindfulness in archery athletes should be strengthened. Athletes who use cognitive reappraisal in archery competition should be aware of their potential appropriation of cognitive resources and should be directed to enhance sport confidence or develop a positive orientation to arouse emotion.

On the other hand, we also find research, such as the one carried out by Bisagno et al., that has focused on finding out which psychological abilities are the most relevant to train, depending on whether it is a sport modality in which open or closed skills predominate or the relationship that exists between the mental abilities of the athlete and the gender and specific position of the game. Thus, the results found by Jakšić et al. demonstrated that Bull's Mental Abilities Questionnaire in the Serbian sample of handball players reflected satisfactory although different psychometric characteristics with the original one. Finally, the study carried out by Török et al. analyzes the perfectionist drive of elite athletes vs. worries as opposite predictors of self-elimination with the mediating role of attributional style. A first step within a broader program aiming to reduce self-enhancement in high-level athletes through attributional retraining intervention.

In the field of health and quality of life of athletes, Wang Y. et al., demonstrated the importance of favoring intrinsic motivation to increase the intention to use mobile technology, such as sports bracelets, which facilitate the acquisition of a healthy lifestyle, Vieira et al. described the profile of fitness professionals and the importance of improving their working conditions and quality of life and Zhang et al. how a meta-analysis shows that although more research is still needed, the results of the studies confirm that physical exercise has moderate positive effects on depression in adolescents. It should be remembered that in the COVID-19 confinement stage there were profound changes in relation to the practice of physical activity that affected the motivation and satisfaction of the basic psychological needs of the athletes, also affecting their social identity, this was demonstrated by Parker et al.. Moreover, LaForge-MacKenzie et al. showed a positive association has been demonstrated between sports practices during the pandemic and low levels of depressive symptoms, i.e., better mental health outcomes in children and young people.

Finally, in the educational context we find two problems that continue to be investigated, on the one hand, to know which are the ideal teaching methods to apply in Physical Education classes to promote intrinsic motivation of students and increase their learning (Ezeddine et al.) and on the other hand, to find the multidisciplinary factors that influence the identification of sports talents at school (Xiang et al.). In the first study, Ezeddine et al. found that the problem-solving method is an effective strategy to improve motor skills and performance, as well as to develop motivation during physical education courses. And in the second, Xiang et al. showed that the main factors affecting sports talent identification in physical education curriculum are personal physical quality, psychological quality, coach's knowledge, and schools' sports talent identification policies.

## Author contributions

All authors listed have made a substantial, direct, and intellectual contribution to the work and approved it for publication.

